# The Prevalence of Functional Gastrointestinal Disorders in the Chinese Air Force Population

**DOI:** 10.1155/2013/497585

**Published:** 2013-04-04

**Authors:** Wenming Wu, Xu Guo, Yunsheng Yang, Lihua Peng, Gaoping Mao, Hyder Qurratulain, Weifeng Wang, Gang Sun

**Affiliations:** ^1^Department of Gastroenterology and Hepatology, Chinese PLA General Hospital, No. 28 Fu Xing Road, Beijing 100853, China; ^2^Department of Gastroenterology and Hepatology, Air Force General Hospital of PLA, No. 30 Fu Cheng Road, Beijing 100142, China; ^3^Department of Gastroenterology-Hepatology, Pakistan Institute of Medical Sciences (PIMS), Islamabad 45320, Pakistan

## Abstract

*Background.* Functional gastrointestinal disorders (FGIDs) are common in the general population worldwide. However, there is paucity of large sale surveys for prevalence of FGID in the military personnel. *Methods.* It is a cross-sectional study, using Rome III criteria for the diagnosis of FGID among the Chinese Air Force (CAF) workers. *Results.* Of 4633 registered male subjects, there were 818 (16.4%) air crew and 4170 (83.6%) ground personnel. FGIDs were identified in 1088 (23.48%) of cases. It was more prevalent in the ground personnel than air crew (24.02% versus 20.33%; *P* = 0.022). Based on Rome III criteria, the commonest disease category was functional gastroduodenal disorder (37.4%), whereas functional nausea and vomiting disorder (FNV) was the most frequent overall diagnosis. Functional dyspepsia (FD) with irritable bowel syndrome (IBS) was the leading FGIDs' overlap (3.9%). *Conclusion.* FGIDs in CAF population are rather underestimated. This necessitates preventive strategies according to job characteristics.

## 1. Introduction

FGIDs comprise idiopathic disorders, characterized by chronic and bizarre complaints arising from dysmotility and hypersensitivity of the digestive system. The diagnosis is made by symptom-based approach using Rome III criteria [1]. These functional disorders render a negative impact on patients' quality of life and induce substantial cost on healthcare system through diagnostic evaluation [2, 3]. The prevalence of FGIDs varies enormously on account of differences in environmental factors, population, and ascertainment criteria. There is considerable overlap between different functional disorders of the digestive tract in general population [4]. This aspect is of special interest in the air force personnel, considering their occupations, arduous working environment, and exposure to various chemicals including jet fuel [5]. These personnel are also subjected to air sickness [6], altitude flying [7], combat stress, and prolonged separation from home [8]. Their job requires an all time high level of physical and mental performance [9]. The scarcity of large scale surveys about FGIDs in military service has prompted us to undertake this study in the CAF personnel. Our aim is to assess the prevalence and overlap of FGIDs in these subjects with a focus on their service requirements.

## 2. Patients and Methods

Between September 2008 and March 2009, a cross-sectional survey was performed in the representative CAF population (*n* = 5800), located at six different regions of China. A randomized, multistage sampling methodology was used for this purpose. All respondents were required to complete a questionnaire, which consisted of two parts: Part 1 pertained to demography, that is, age, marital status, ethnicity, education, and job description; Part 2 was a modified Chinese version of the FGIDs self-report questionnaire (FGIDs-Q) based on Rome III criteria and comprising 93 questions [10]. The sampled population was interviewed by squadrons' flight surgeon and the information was recorded on a computer using software EPIDATA3.02. The present study was approved by Institutional Review Board of the Chinese People's Liberation Army General Hospital. Written informed consent was obtained from all participants.

Statistical analysis was performed with SPSS software version 13.0 (SPSS Inc, Chicago, IL, USA). Chi-square test and Fisher exact test were used for detecting significant differences in the percentage of categorical data. *P* value <0.05 was taken as statistically significant.

## 3. Results

### 3.1. Demographic, Social, and Military Characteristics of the CAF Population

Our questionnaires were duly completed and returned by 5423/5800 of subjects (i.e., response rate 93.6%). The participants demonstrated normal health, mean age 23.6 years (range: 16 years to 53 years), body mass index (BMI) 17 to 33, and mean years of military service 4.2 years (range: 0.6 years to 32 years). Of 4988 (92.0%) qualified respondents to the first questionnaire, air crew were 818 (16.4%) and ground personnel were 4170 (83.6%). No racial disparity was appreciable between the two groups (Table 1). However, significantly more officers were included in the aircrew than ground personnel (92.4% versus 7.5%; *P* < 0.000) with higher education (graduation: 94.6% versus 33.2%; *P* < 0.001) and longer years of service (>16 years: 26.8% versus 2.8%; *P* < 0.000) evident in the air crew. Conversely, the majority of eligible cases were ground personnel (92.5% versus 7.4%; *P* < 0.001), single (38.7% versus 77.6%), young (<25 and, years: 68.9% versus 28.4%).

### 3.2. Spectrum of the FGID

**Figure 1:**
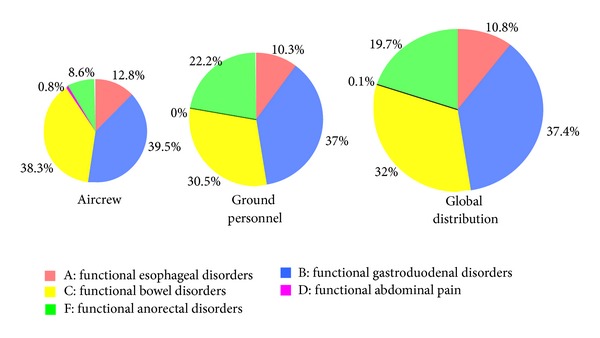
Distribution of 5 major categories (A to F) of functional gastrointestinal disorders (FGIDs) according to Rome III criteria.

From a total of 4988 initial responders, 4633 of the participants, including 787 (17.0%) aircrew and 3846 (83.0%) ground personnel, were eligible for the current analysis on submitting FGIDs questionnaire. More than one FGID were identified in 1088 (23.5%) of subjects: ground personnel 928 (85.3%) and aircrew 160 (14.7%). The ground personnel also showed greater prevalence of FGID than aircrew (24.12% versus 20.33%; *P* = 0.022). We observed distribution of 5 major categories of FGIDs (no subject with category E biliary disorders was found in this survey) based on Rome III criteria: functional gastroduodenal disorders (FGD: 37.4%), functional bowel disorders (FBD: 32.0%), functional anorectal disorders (FAD: 19.7%), functional esophageal disorders (FED: 10.8%), and functional abdominal pain (FAB: 0.1%) (Figure 1).

The prevalence of individual FGID in the present study is as follows. Nausea and vomiting disorders (NVD: 6.93%), functional abdominal bloating (FAB: 6.39%), functional dyspepsia (FD: 5.85%), and irritable bowel syndrome (IBS: 4.04%). Functional heartburn (FH: 1.38%) was the most frequent diagnosis amongst functional esophageal disorders, whereas functional anorectal pain (FAP: 3.78%) and functional defecation disorder (FDD: 2.35%) led the functional anorectal disorders (Table 2). On comparing prevalence by *χ* ^2^ statistics for the aircrew and ground personnel, significant differences were appreciable in the prevalence of various FGID: FH (2.41% versus 1.17%; *P* = 0.006), NVD (5.08% versus 7.31; *P* = 0.025), IBS (5.72 versus 3.07%; *P* = 0.09), functional constipation (FC: 2.03% versus 0.75%; *P* = 0.001), and functional incontinence (FI: 0.38% versus 2.76%; *P* ≤ 0.001).

### 3.3. Overlap between FGIDs Categories

**Figure 2:**
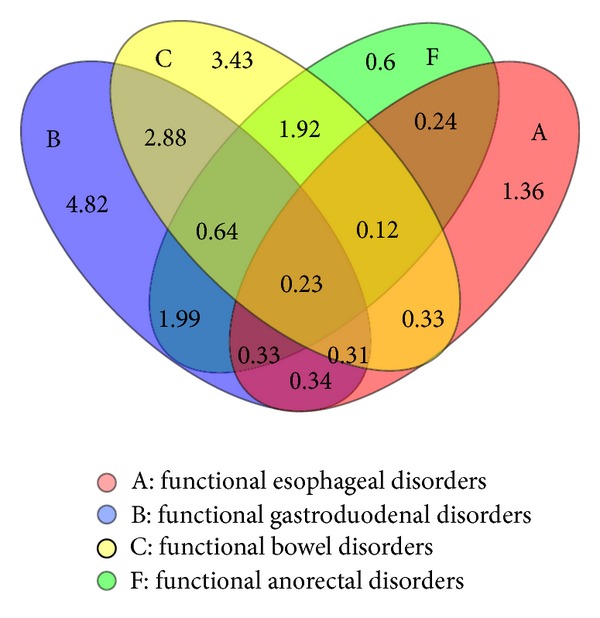
Venn diagram demonstrating overlap between functional esophageal, gastroduodenal, bowel and anorectal disorders in the study population based on Rome III criteria. The values correspond to prevalence (%) within a subset.

A combination of various FGIDs was evident in 615 (13.27%) of our cases, whereas another 473 (10.21%) suffered from only one group of disorder. Venn diagram shows the overlap between four defined categories of FGIDs according to Rome criteria III: category A (functional esophageal disorders), category B (functional gastroduodenal disorders), category C (functional bowel disorders), and category F (functional anorectal disorders). The values represent prevalence (%) in each category: category A alone 1.36%, categories A and C alone 0.33%, categories A, B and C alone 0.31%, categories A, B, C and F 0.23%. No striking pattern was observed (Figure 2).

The categories of FGIDs occurred in different combinations in our air force workers: two-way combination 0.92%–4.06%, three-way combination 0.35%–0.87%, and four-way combination 0.23%. Categories B and C of FGIDs were the most prevalent categories in these complexes. FD-IBS 2.89% was the most frequent combination whereas four-way complexes, comprising FH + FD + IBS + FAP, constituted only 0.08% of persons (Table 3).

## 4. Discussion

This is the first population-based military survey using Rome III criteria to evaluate FGIDs in the CAF workers. According to our observation, FGIDs is a rather low prevalent functional disorder in healthy military personnel. On the contrary, prevalence of FGIDs in the Chinese citizens ranges from 5.67% to 55.24% [11, 12]. There are several possible explanations for this difference in the prevalence of FGIDs. The most plausible reason may be the induction of a male predominant group of respondents in this survey. Our participants are healthy military men, who do not represent the real Chinese general population. The gender predilection in majority of FGIDs exists. There is an obvious female predominance on account of psychosocial factors and difference in the hormonal ratios [13]. The diversity in prevalence rates may also be due to wide variation in study designs and sampling methods.

The majority of aircrew in this survey are pilots. It is not clear why the aircrew reported slightly lesser FGID than ground personnel. However, we presume that pilots mainly belong to the officer class, who possess higher level of technical education than workers in the support services. The job of aircrew requires a highly efficient and error-free performance. They undergo regular physical and psychiatric evaluation by the flight surgeon for routine flying and flight training. Only qualified candidates with higher psychophysical score are selected as aircrew [14]. On the other hand, ground personnel include aeronautical engineers, fuel technicians and airfield defense troops. The enlisted ranks normally constitute a massive proportion of the ground personnel. They require lower formal education according to the occupational needs. The ground personnel are required to perform arduous tasks. They enjoy lesser autonomy and personal control as compared with the aircrew. Thus FGIDs symptoms amongst ground workers are apparently caused or aggravated by tough working environment, adverse psychological features, and nongastrointestinal conditions [15]. However, more studies should be carried out to determine the impact of specific occupational, organizational, and psychosocial factors on the clinical presentation of FGIDs.

Our results indicate that FNV was the most prevalent FGIDs overall and in the ground personnel. It was also the second most frequent functional disorder in aircrew. On account of low prevalence of these symptoms, there is no convincing explanation in the medical literature to support the underlying mechanism for FNV in the civilian population [16]. Several trigger factors can be found in approximately 80% of patients with cyclic vomiting syndrome [17]. They include infection, psychological stress, motion sickness, menstruation, lack of sleep, and physical exhaustion [18]. The triggers appear to be more common in a military setting due to increased stress of flying sophisticated combat aircraft. A skilful handling of these complicated machines involves high speed flying, constant exposure to rapid acceleration/deceleration (i.e., “g” forces), vibration, noise pollution, spatial disorientation and risk of rapid decompression [19]. Nausea and vomiting are common manifestations of high altitude flying [20, 21]. Motion sickness, nausea, and vomiting are also caused by combined lateral and roll oscillation during flying [22, 23]. The aerotechnicians are at risk of developing the above symptoms when subjected to irritant jet fuel during accidental spills, degreasing, and fuel storage. The aeronautical engineers are also at similar risk during general maintenance or operation of the military aircraft and vehicles. The data for epidemiological survey of the Spanish Ministry of Health & Consumer Affairs shows that nausea and vomiting were major complaints by the workers engaged in clean-up of the Prestige oil-spill [24]. However, few related manuscripts are available about the influence of these factors on FNV in specific settings because the exact underlying mechanism for this finding remains to be established.

As shown in the previous studies, several other highly prevalent FGIDs, including IBS, FD, and FAB, are associated with diverse pathophysiological mechanisms. The data of Defense Medical Surveillance System (1999–2007) describes the distribution of main FGID as FC 55%, dyspepsia 21.2%, FD 2.1%, IBS 28.5% [25]. The ratio of FC and IBS in this data is higher than our study. The reason for this incompatibility may be due to more subcategories of FGID. It is also suggested that infectious gastroenteritis during deployment increases the risk of FGID in these workers [26]. The same system further suggests that dysmotility may result from deployment-related travellers' diarrhea although these findings need to be confirmed. According to them, lower military ranks are more vulnerable to develop IBS (OR: 3.70; *P* = 0.02) [26]. This observation is consistent with our results. Besides infection, noise and other occupational exposures have significant association with IBS [27]. The available evidence indicates that aircraft noise increases IBS prevalence in residents around the military airbase in Pyeongtaek city [28].

Several studies demonstrate that FGIDs' overlap is a common occurrence [29, 30]. The prevalence rates for different combinations of FGIDs range from 0.23% to 4.06% in our survey. These figures appear small but the overall rate of FGIDs complexes actually accounts for >50% of total participants in the present study. This observation is coincident with a previous survey in the civilian population [29]. Some studies advocate that various combinations of FGIDs may result from a common pathogenesis [30]. Psychosocial abnormalities seem to play an important role in the development or exacerbation of FGIDs' overlap. Depression, anxiety, and posttraumatic stress disorder tend to favor the development of IBS with dyspepsia in women receiving primary care at the Veteran Affairs Medical Center. The aforementioned mechanisms thus provide some clue to high incidence of overlap in a particular setting.

There are some limitations in the current survey. Our sample was derived from frontline troops. The females could not be included due to their small percentage in the CAF population. The study was based on self-reported data so that organic diseases were not ruled out in our subjects.

## 5. Conclusion

In summary, our study provides an objective evidence to examine the impact of several military service-related factors on the prevalence of FGIDs. The preventive strategy should be rationally planned according to the occupational characteristics.

## Figures and Tables

**Table 1:** Demographic, social, and service characteristics of the Chinese air force personnel, responding the first questionnaire: (*n* = 4988).

Variables	Aircrew (*n* = 818)	Ground personnel (*n* = 4170)	*P* value
Age			<0.001
<25	232 (28.4)	2911 (69.8)	
25–30	242 (29.6)	951 (22.8)	
>35	344 (42.0)	308 (7.4)	
Race			0.16
Han race	793 (96.9)	3999 (95.9)
Minority	25 (3.1)	171 (4.1)
Marital status			<0.001
Single	317 (38.7)	3236 (77.6)	
Married	465 (56.8)	746 (17.9)	
Separated/divorced	36 (4.5)	188 (4.5)	
Education			<0.001
Less than high school	1 (0.1)	575 (13.8)	
High school	30 (3.7)	2144 (51.4)
College graduate	774 (94.6)	1384 (33.2)
Postgraduate	13 (1.6)	67 (1.6)	
Years of service			<0.001
<4	120 (14.7)	2052 (49.2)	
4–16	479 (58.5)	2001 (48.0)	
>16	219 (26.8)	117 (2.8)	
Rank			<0.001
Officer	757 (92.4)	313 (7.5)
Enlisted	61 (7.4)	3857 (92.5)

**Table 2:** The prevalence of functional gastrointestinal disorders (FGIDs) using Rome classification and the modified questionnaire.

Functional gastrointestinal disorders	Aircrew (*n* = 787)	Ground personnel (*n* = 3846)	*P* value	Total (*n* = 4633)
A. Esophageal disorders	34 (4.32)	120 (3.12)	0.087	154 (3.26)
A1: globus	9 (1.14)	30 (0.78)	0.309	39 (0.84)
A2: functional chest pain	6 (0.76)	40 (1.04)	0.474	46 (0.99)
A3: functional heartburn*	19 (2.41)	45 (1.17)	0.006	64 (1.38)
A4: functional dysphagia	1 (0.13)	10 (0.26)	0.419	11 (0.24)
B. Gastroduodenal disorders	105 (13.34)	430 (11.2)	0.084	535 (11.54)
B1: functional dyspepsia	50 (6.35)	221 (5.75)	0.509	271 (5.85)
B2: belching disorders	22 (2.80)	97 (2.72)	0.659	119 (2.57)
B3: nausea and vomiting disorders*	40 (5.08)	281 (7.31)	0.025	321 (6.93)
B3a: chronic idiopathic nausea*	21 (2.67)	71 (1.84)	0.024	92 (1.99)
B3b: functional vomiting*	1 (0.13)	28 (0.87)	0.031	29 (0.62)
B3c: cyclic vomiting syndrome*	23 (2.92)	191 (4.97)	0.013	214 (4.62)
B4: rumination syndrome in adults	4 (0.51)	18 (0.47)	0.999	22 (0.47)
C. Bowel disorders*	102 (12.96)	355 (9.10)	0.001	457 (9.86)
C1: irritable bowel syndrome*	45 (5.72)	142 (3.70)	0.009	187 (4.04)
C2: functional abdominal bloating	68 (8.64)	228 (5.93)	0.005	296 (6.39)
C3: functional constipation*	16 (2.03)	29 (0.75)	0.001	45 (0.97)
C4: functional diarrhea	15 (1.91)	56 (1.46)	0.349	71 (1.53)
D. Functional abdominal pain	2 (0.27)	0 (0.00)	NC^∧^	2 (0.05)
F. Anorectal disorders*	23 (2.92)	258 (6.71)	0.000	281 (6.07)
F1: functional incontinence*	3 (0.38)	106 (2.76)	0.000	109 (2.35)
F2: functional anorectal pain	22 (2.80)	153 (3.98)	0.113	175 (3.78)
F2a: levator ani syndrome	2 (0.27)	21 (0.55)	0.434	23 (0.50)
F2b: proctalgia fugax	20 (2.54)	134 (3.48)	0.179	154 (3.32)

^#^Subjects may have more than one group of disorders.

*P < 0.05 aircrew versus ground personnel.

^∧^NC: not compared.

**Table 3:** The prevalence of overlap in functional gastrointestinal disorders (FGIDs).

Two-way combination	*N* (%)
B + C	**188** (**4.06%**)
FD + IBS	134 (2.89%)
Others	54 (1.17%)
B + F	** 148** (**3.19%**)
NVD + FAP	64 (1.38%)
Others	84 (1.81%)
C + F	**135** (**2.91%**)
IBS + FAP	45 (0.97%)
Others	90 (1.94%)
A + B	**56** (**1.21%**)
FH + FD	17 (0.37%)
Others	39 (0.84%)
A + C	** 46** (**0.99%**)
FAB + FC	15 (0.32%)
Others	31 (0.67%)
A + F	** 43** (**0.92%**)
FCP + FI	13 (0.28%)
Others	30 (0.64%)

Three/four-way combination	

B + C + F	**40** (**0.87%**)
IBS + FD + FAP	16 (0.34%)
Others	24 (0.53%)
A + B + F	**24** (**0.56%**)
FH + FD + FI	13 (0.29%)
Others	10 (0.27%)
A + B + C	**25** (**0.54%**)
FH + NVD + IBS	11 (0.23%)
Others	14 (0.31%)
A + C + F	**16** (**0.35%**)
FCP + FC + FI	9 (0.19%)
Others	7 (0.16%)
A + B + C + F	**11** (**0.23%**)
FH + FD + IBS + FAP	4 (0.08%)
Others	7 (0.15%)

FCP: functional chest pain, FH: functional heartburn, FI: functional incontinence, IBS: irritable bowel syndrome, FAB: functional abdominal bloating, FAP: functional anorectal pain, and NVD: nausea and vomiting disorders.
